# Ethnobotanical survey, anthelmintic effects and cytotoxicity of plants used for treatment of helminthiasis in the Central and Kara regions of Togo

**DOI:** 10.1186/s12906-020-03008-0

**Published:** 2020-07-07

**Authors:** Essoham Ataba, Gnatoulma Katawa, Manuel Ritter, Adjoa Holali Ameyapoh, Kokou Anani, Oukoe M. Amessoudji, Pélagie Edlom Tchadié, Tchadjabo Tchacondo, Komlan Batawila, Yaovi Ameyapoh, Achim Hoerauf, Laura E. Layland, Simplice D. Karou

**Affiliations:** 1 Ecole Supérieure des Techniques Biologiques et Alimentaires (ESTBA)/Laboratoire de Microbiologie et de Contrôle de Qualité des Denrées Alimentaires/Unité de Recherche en Immunologie et Immunomodulation (UR2IM), Université de Lomé, 01 BP 1515 Lomé, Togo; 2 Institute for Medical Microbiology, Immunology and Parasitology (IMMIP), University Hospital Bonn (UKB), Bonn, Germany; 3 Laboratoire de Biologie et Ecologie Végétale, Faculté des Sciences (FDS), Université de Lomé, Lomé, Togo; 4 German Center for Infection Research (DZIF), partner site Bonn-Cologne, Bonn, Germany

**Keywords:** Anthelmintic effects, Ethnopharmacology, Medicinal plants, Togo

## Abstract

**Background:**

Traditional medicines are the main source of treatment of helminthiasis in endemic areas of Togo. The present study aimed to investigate the plants used by Traditional healers (THs) to treat helminth infections in endemic communities within the Central and Kara regions of Togo and to evaluate the anthelmintic activity of the three most cited plants.

**Methods:**

An ethnobotanical survey was conducted from 19 to 24 June 2017 among traditional healers in the Central and Kara regions of Togo. The anthelmintic activity of the most cited plants namely *Aframomum melegueta* K. Schum, *Khaya senegalensis* A. Juss and *Xylopia aethiopica* A. Rich, was evaluated using microfilariae (Mf) of *Litomosoides sigmodontis*. The plants were evaluated for cytotoxicity according to the recommendation of NF EN ISO 10993-5 standard using the propidium iodide (PI) dye by flow cytometry on human peripheral blood mononuclear cells.

**Results:**

A total of 197 THs were interviewed and 41 plant species were recorded. Leguminosae (14.6%) and Annonaceae (9.7%) families constitute the highest number of species cited for treatment of helminth infections. *Afromomum melegueta* was the most cited by the THs for the treatment of onchocerciasis (UV = 0.036) while *X. aethiopica* was associated with the treatment of schistosomiasis (UV = 0.061) and lymphatic filariasis (UV = 0.061). There was a great agreement among the THs regarding ethnomedicinal uses of plants to treat helminthiasis with ICF values ranging from 0.57 to 0.67. The anthelmintic assay yielded lethal doses values of 233 μg/mL, 265 μg/mL and 550 μg/mL, respectively for *X. aethiopica*, *A. melegueta* and *K. senegalensis*. *Afromomum melegueta* and *X. aethiopica* presented no cytotoxicity, less than 20% death, whereas *K. senegalensis* induced moderate toxicity, 24 ± 8% death.

**Conclusion:**

This study demonstrated the scientific rationale for the use of plants to treat helminthiasis in the Togolese traditional medicine. However, the use of *K. senegalensis* requires more caution since the plant is fairly toxic.

**Trial Registration:**

**NA**

## Background

The emergence of resistance to anthelmintics makes it difficult to control helminth infections in endemic areas. One of the solution approaches is the search for new molecules and the development of effective therapies, affordable for low-income people, since the populations affected are leaving in developing countries. Many studies are already devoted to this, and even a new method of screening for filaricidal agents has recently been developed [1]. In this new approach, plants from the traditional pharmacopoeia have demonstrated proven anthelmintic effects [2].

Herbal medicines have been the source of many of the drugs prescribed today in modern medicine. Some examples are, aspirin from *Salix alba* [3], digitoxin from *Digitalis* [4], artemisinin from *Artemisia annua* [5]. Medicinal plants are precious resources in low-income countries and more than 80% of African populations use them for health problems [6]. Therefore, the use of plant organs to heal is a question of culture and tradition in Africa [7, 8]. Understanding the properties and value of unprocessed raw medicinal plant materials is a national heritage for these countries [9]. It should be noted that for primary health care needs, a large part of the African population still turns to traditional medicine which is mainly based on herbal remedies. This is due in part to the preference and confidence of local healers over the health care system [10].

Chronic infections with helminths namely *Onchocerca volvulus*, *Wuchereria bancrofti* and *Schistosoma haematobium* induce diseases in endemic areas of Togo. The absence of vaccines, the constant exposure and the possibilities of reinfection with these helminths present a constant socio-economic problem and an increase in DALYs (disability-adjusted life years). Togo has an excellent biodiversity of medicinal plants used in traditional medicine for the treatment of many diseases. Thus, many traditional remedies have been developed by the practitioners of traditional medicine to treat helminth infections. However, scientific data on these herbal therapies are missing. The present study was initiated with the aim of documenting the plants usage in the treatment of helminth infections by traditional healers (TH) in endemic communities in the Central and Kara regions of Togo and in assessing their anthelmintic and cytotoxic effects in vitro.

## Methods

### Study area

**Fig. 1:**
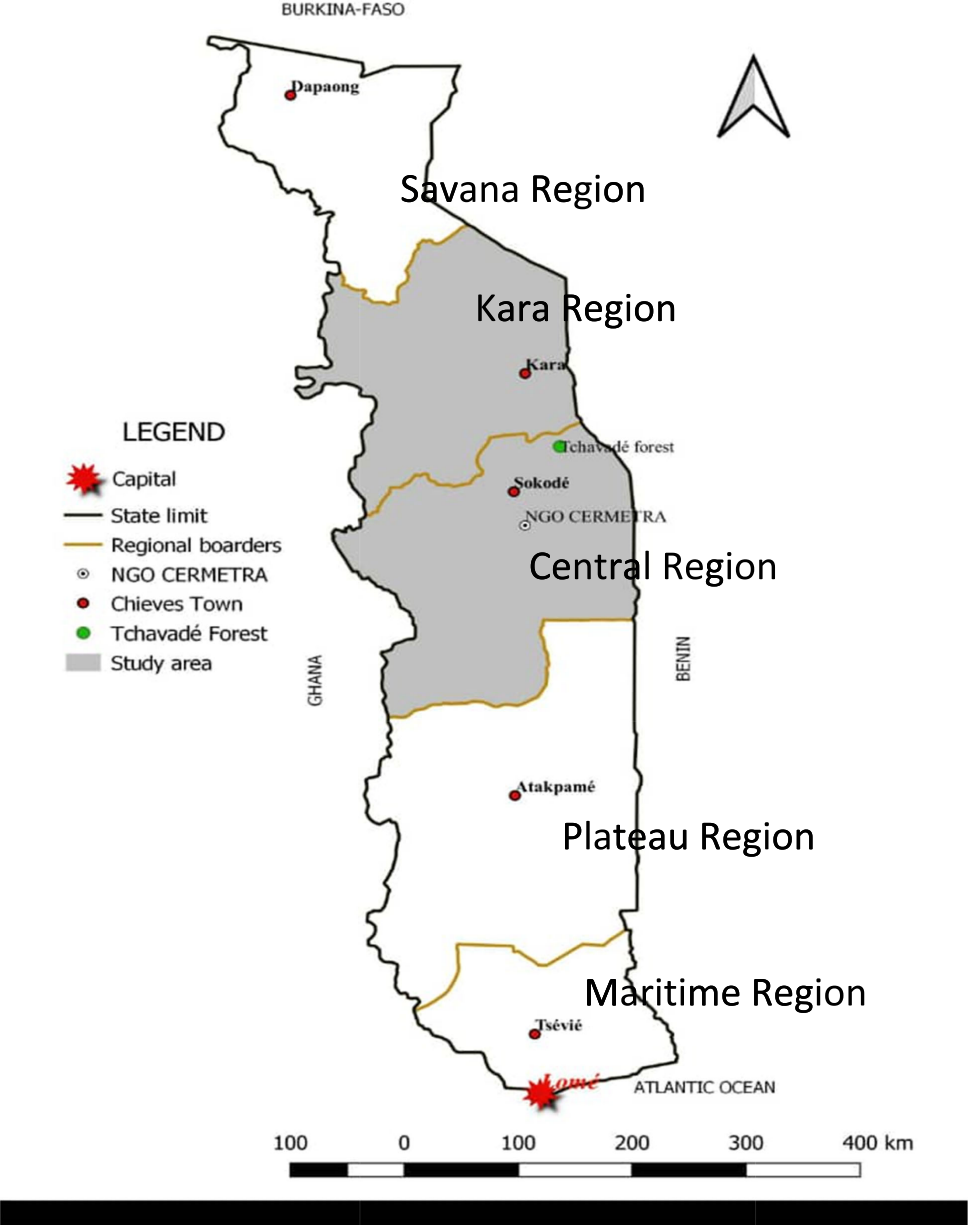
Map of Togo showing the study area. The study was conducted in the Central and Kara regions of Togo. THs usually gather plant species for their medicines in the Tchavedè Forest. Various plant organs were collected for botanical identification

The ethnobotanical survey was undertaken in the Central and Kara regions of Togo (Fig. 1). Togo is a West African country boarded in the North by the Republic of Burkina Faso, the Est. by the Republic of Benin, the West by the Republic of Ghana and the South by the Atlantic Ocean. From north to south the country is organized into five economic regions: the Savannah region, the Kara region, the Central region, the Plateaux region and the Maritime region. The Central and Kara regions belong to the tropical area with a dry season from October to March and a rainy season from April to September. The annual temperatures are between 20 and 39 °C, providing an excellent floristic biodiversity with numerous medicinal plants. The principal activities of the population are agriculture and trade [10].

### Data collection

In total, 197 THs (136 from central region and 61 from Kara region) were interviewed from the 19th to 24th June 2017 using a structured questionnaire, after their informed consent and their agreement with a signature. The THs belong to the *Tem* tribe and *Kabyè* tribe, and all of them speak at least one local language, *Kotokoli* or *Kabyè* in which interviews were conducted. They were all members of the non-governmental organization (NGO) named “Centre d’Etude et de Recherche en Médecine Traditionnelle Appliquée du Togo” (CERMETRA) (http://tg.viadeo.com/fr/profile/cermetra.ong). CERMETRA contributes to the training and counselling of the THs on patient management and environmental preservation, mainly protecting vulnerable and endangered plant species used in traditional medicine. For example, the harvest of the leaves of *Pterocarpus erinaceus* which is a species included in the red list of the IUCN (https://www.iucnredlist.org/species/62027797/62027800), is rigorously supervised by CERMETRA after the authorisation of the forestry services to avoid the removal of vital organs such as stem bark and roots. The main activity that threatens extinction *P. erinaceus* is the exploitation of its wood, it is not really the use in traditional medicine, which moreover focuses mainly on the leaves and not the vital organs. Actions carried out in the country therefore aim to limit the exploitation of its wood and reforestation. Thus, the University of Lomé through the Faculty of Sciences is very active in revitalizing the populations of *P. erinaceus*. The University is a partner of the “South Expert Plants Sustainable Development” (SEP2D) multilateral program. The challenge of this program is to strengthen interactions and partnerships in terms of plant biodiversity between research, teaching and society’s demands. In the particular case of *P. erinaceus*, proposals have been made in order to provide the elements of responses essential to the adoption of sustainable silvicultural practices which should allow a rapid reconstitution of populations in West Africa. It specifically involves (i) analyzing the biophysical and socio-cultural factors of the multiplication and domestication of *P. erinaceus* in 3 countries in West Africa (Togo, Benin and Niger), (ii) studying the variability in the structural and technological characteristics of the species’ wood in relation to environmental conditions, (iii) developing a viable and large-scale production strategy for *P. erinaceus* plants for reforestation in West Africa and (iv) strengthen the capacities of stakeholders to regenerate and sustainably manage stands of *P. erinaceus*. http://www.sep2d.org/projets-soutenus/recherche-operationnelle/reconstitution-peuplement-pterocarpus-togo-benin-niger.

After the interview, 41 of the most commonly plant species used by THs to treat helminthiasis were collected. Since the THs often referred to these plants in a colloquial manner, samples of all 41 plants were collected with members of CERMETRA (actually THs) in the Tchavadè forest. Verification and identification of all collected plant specimens were carried out at the Botanic Laboratory of the Faculty of Sciences at the University of Lomé. Plant taxonomy was confirmed on data available from the International Plant Names Index (IPNI) website: http://www.ipni.org/. Specimen of each plants was deposited at the herbarium of the University of Lomé.

### Preparation of plants extracts

All the plants materials were collected from the Tchavadè forest at Sokodé in the central Region with Traditional healers. The grains of *A. melegueta*, bark of *K. senegalensis* and fruits of *X. aethiopica* were washed and air-dried in laboratory at room temperature. Plant materials were reduced to powder that was used for extraction. The extraction was performed by percolation of 100 g powder with 500 mL ethanol-water (70: 30) for 48 h. The extract was then filtered with Whatman paper and the filtrate was evaporated until dry using a Rotary evaporator at 50 °C under reduced pressure.

To perform cytotoxicity and anthelmintic tests, 100 mg/mL extracts were prepared by dissolving 1 g of dried extract in 10 mL distilled water. From this, serial dilutions were made and filtered using 0.45 μm millipore adapted to a syringe.

### Purification of peripheral blood microfilariae (mf)

Frozen Mf were obtained from the Institute for Medical Microbiology, Immunology and Parasitology, University Hospital Bonn (UKB), Bonn, Germany. The Mf for in vitro anti-microfilarial test were isolated from the peripheral blood of *L. sigmodontis*-infected cotton rats [11]. Blood was diluted with PBS in the ratio 1:2 and carefully loaded onto a 30–25% Percoll gradient (Sigma-Aldrich GmbH, Munich, Germany). After 30 min centrifugation (300 g) at room temperature without break, the recovered Mf were washed two times with non-supplemented RPMI-1640 medium (PAA, Linz, Austria), counted and frozen at − 80 °C in freezing medium containing 6% DMSO and 15% fetal calf serum (FCS) (PAA). For this experiment, fresh aliquots of frozen Mf were thawed and controlled for Mf viability microscopically after 2 h of pre-incubation at 37 °C in RPMI-1640 medium containing 10% FCS in order to revitalize the microfilariae. Only aliquots with more than 95% Mf viability were used.

### Anthelmintic assay

For the assay, 100 μL/well of suspension containing 75 microfilariae were grown in RPMI 1640 in a 96-well plate with plant extracts 200, 500 and 1000 μg/mL. The control was a subculture without plant extract. Albendazole (5 mg/mL) was used as reference drug as previously described [2]. After 7 days incubation at 37 °C in a humid atmosphere, the viability of the microfilariae was evaluated microscopically using trypan blue exclusion method. Living and dead Mf were counted for each concentration of drug. The concentration that induced 50% Mf death was considered as LD_50_.

### Cytotoxicity assay

The cytotoxicity assays was conducted according to the recommendation of NF EN ISO 10993-5 standard. Human peripheral blood mononuclear cells from healthy volunteers (*n* = 13) were isolated using ficoll density gradient centrifugation method and treated with 200 μg/mL plant extracts for 24 h at 37 °C under 5% CO_2_. Afterwards, cell pellets were harvested and stained with propidium iodide (PI) dye prior to cell acquisition using Cytoflex flow cytometer (Beckman Coulter, Brea, USA). From the lymphocytes gate, the percentage of cells expressing propidium iodide (PI) was assessed. Data were analysed using CytExpert 2.1 sofware (Beckman Coulter, Brea, USA).

### Data analysis

A Microsoft Excel spreadsheet 2013 was used to perform simple calculations and determine plant frequencies. The relative importance of species was evaluated by the frequency in which it was mentioned by the THs and a “Used value” (UV) was calculated as follows: UV = ΣU/*n* (ΣU is the total number of citations per species and *n* is the number of interviewed THs. The UV is useful in determining which plants have the best use and are most often indicated in the treatment of a disease [12]. The agreement (Informant consensus factor (ICF)) of the THs regarding the uses of medicinal plants to treat helminthiasis was calculated by the following formula: ICF=Nuc -Nt/(Nuc − 1) where Nuc is the number of citations for the treatment of a given disease and Nt is the number of species used in the treatment of a given disease [13].

Statistical analyses were performed using Graph Pad PRISM 5.02 software (GraphPad Software, La Jolla, USA). The χ^2^ test was used for the comparison between groups and the difference was considered significant with a *p*-value< 0.05.

## Results

### Ethnobotanical study

#### Characteristics of traditional healers

A total of 197 THs were interviewed, among them 168 were men and 29 were women. 76.14% of the cohort were older than 40 years and 77.66% practiced traditional medicine for more than 10 years (Table 1). The majority of THs inherit the knowledge from their family according to the percentage in that category (77.16%). According to the ethnical affiliation, the interviewed THs belonged to *Tem* (82.65%), *Mina* (6.64%), *Moba* (8.67%) and *Gourma* (2.04%) tribes.

**Table 1:** Characteristics of THs treating Helminthiasis

	Gender ***N***(%)	Age ***N*** (%)		Experience ***N*** (%)			Origin of the knowledge ***N*** (%)		
		< 40 years	≥ 40 years	0–4 years	5–9 years	≥ 10 years	Heritage	Training	Calling
**Men**	168(85.28)	38(80.85)	130(8.67)	10(55.56)	22(84.62)	136(88.89)	133(67.51)	21(10.66)	14(07.10)
**Women**	29(14.72)	9(19.15)	20(13.33)	8(44.44)	4(15.38)	17(11.11)	19(9.64)	9(04.57)	1(0.51)
**Total**	197 (100)	47(23.86)	150(76.14)	18(9.14)	26(13.20)	153(77.66)	152(77.16)	30(15.22)	15(7.61)

#### Main Helminthiasis treated by THs

**Fig. 2:**
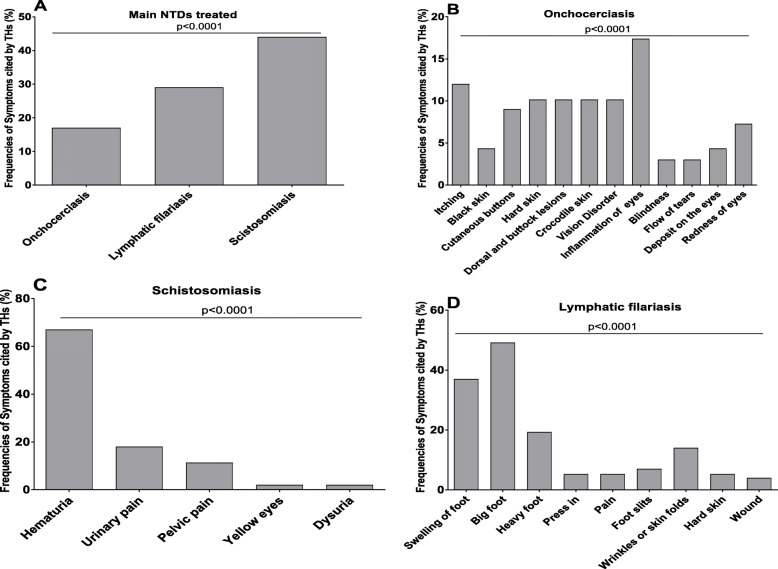
Frequencies of helminthiasis treated and diagnosis by the THs. Bar represents the percentage of THs (*n* = 197) treating suspected cases of onchocerciasis, lymphatic filariasis, schistosomiasis, dermatitis and inflammation **a** and symptoms used by the THs (=197) for the diagnosis of the diseases: onchocerciasis **b**, schistosomiasis **c** and lymphatic filariasis **d**. Chi-square was used to compare differences between groups and *p* value was less than 0.0001

THs claimed to treat onchocerciasis, lymphatic filariasis and schistosomiasis based on observed clinical symptoms and all interviewed THs were familiar with these three helminth infections. Figure 2a shows the frequencies in which THs had cases and treated the associated pathologies. 43.65% of THs had treated schistosomiasis, 28.93% had treated lymphatic filariasis and 17.26% had treated onchocerciasis.

The THs recognized the disease by the main characteristics of symptoms. Figure 2 shows the list and frequencies of symptoms used by the THs to diagnose onchocerciasis, lymphatic filariasis and schistosomiasis. Itching and eye disorders were the most common symptoms associated with *O. volvulus* infections (Fig. 2b). Hematuria was the most obvious symptom for *S. haematobium* infections with nearly 70% of THs associating this symptom with the infection (Fig. 2c). Lymphatic filariasis (LF) caused by *W. bancrofti* was diagnosed by “big foot” (49%) and “swelling of the foot” (36.84%).

#### Plants used for the treatment of Helminthiasis

A total of 41 plant species from various families were prominently mentioned by THs during the interviews. Most belonged to Leguminosae (14.6%) and Annonaceae (9.7%) families and are listed in Table 2.

**Table 2:** Diversity of plants used for the treatment of helminthiasis: administration route, parts and the mode of preparation

*Species*	Family	Local name	Voucher number	Used parts	Formulation	Route	Use value		
							Onchocerciasis	Lymphatic filariasis	Schistosomiasis
*Acacia sieberiana* Tausch.	Leguminosae	Bovom	TOGO15381	Root	Powder	Oral		0.005	
*Aframomum melegueta* K.Schum.*	Zingiberaceae	Abaltchangai/ Kalmboa	TOGO15382	Seed	Decoction, Dough	Oral, Topical	0.030	0.036	0.025
*Anacardium occidentale* L*.*	Anacardiaceae	Atcha	TOGO15383	Bark	Decoction	Oral			0.005
*Annona senegalensis* Pers*.*	Annonaceae	Tchoyhodè/ Tchutchudè	TOGO15384	Root, Leaves, Bark	Decoction	Topical		0.015	0.005
*Biophytum petersianum* Klotzsch	Oxalidaceae	Kpirikpozo	TOGO15385	Whole plant	Decoction	Oral			0.005
*Blighia sapida* K.D.Koenig	Sapindaceae	Kpézou/ Kpèzéou	TOGO15386	Bark, Fruit, Seed, Leaves	Dough, Decoction	Oral, Topical	0.005		0.010
*Bombax costatum* Pellegr. & Vuillet	Bombacaceae	Folo	TOGO15387	Bark, Leaves	Decoction	Topical		0.005	0.005
*Calotropis procera (Aiton*) W.T.Aiton	Asclepiadaceae	Tchovow	TOGO15388	Leaves, Bark, Root, Sap	Powder	Topical		0.005	
*Cyathula prostrata* Blume	Amaranthaceae	Amatamata	TOGO15392	Leaves	Powder, Decoction	Topical			
*Dychrostachys cinerea* R.Vig.	Leguminosae	Sozozi	TOGO15393	Leaves	Dough, Decoction	Topical			
*Erythrina senegalensis* A.Rich*.*	Leguminosae	Gbingbintoukoloko/ Kpodjkpalo	TOGO15394	Root	Decoction	Oral	0.005		0.010
*Flueggea virosa* Baill.	Euphorbiaceae	Tchakatchaka	TOGO15397	Root, Leaves	Infusion, maceration, Decoction, Powder	Oral, Topical		0.005	0.005
*Hannoa undulata* Planch*.*	Simaroubaceae	Dgbéré	TOGO15398	Root, Bark	Infusion, Decoction	Oral, Topical		0.005	
*Héliotrope indicum* L*.*	Boraginaceae	Soudjondjon, Soukoudjo	TOGO15399	Root	Powder	Oral, Topical			0.010
*Hexalobus monopetalus* Engl. & Diels	Annonaceae	Barakoundou	TOGO15400	Root	Infusion, Decoction Powder	Oral, Topical			
*Jatropha curcas* L*.*	Euphorbiaceae	Sawkofolmo	TOGO15401	Leaves, Sap	Decoction	Oral	0.005		
*Khaya senegalensis* A.Juss.*	Meliaceae	Frémou/ Hèmo	TOGO15402	Bark, Root, Leaves	Dough	Topical, Oral	0.020	0.015	0.036
*Kigelia africana (*Lam.) Benth.	Bignoniaceae	Abiliou/ Limié	TOGO15403	Bark, Leaves, Root, Seed	Mashing	Oral, Topical	0.005	0.020	
*Landolphia hirsuta* (Hua) Pichon	Apocynaceae	Low	TOGO15404	Root	Powder	Oral			0.005
*Lannea barteri* Engl*.*	Anacardiaceae	Kpatandew	TOGO15405	Bark	Decoction, Infusion	Oral			0.015
*Lawsonia inermis* L*.*	Lythraceae	Lali	TOGO15406	Leaves	Powder	Oral			
*Lophira lanceolata* Tiegh. ex Keay	Ochnaceae	Kparakpara	TOGO15407	Leaves, Root	Dough	Topical	0.005		
*Ocimum canum* Sims en u	Lamiaceae	Kozossognna/ Hagzao, Kosonsong	TOGO15409	Leaves, Root, Whole plant	Mashing	Ocular, Oral	0.010	0.005	0.005
*Parkia biglobosa* (Jacq.) R.Br. ex G.Don	Leguminosae	Solo	TOGO15410	Bark, Leaves, Root, Seed	Powder	Oral, Topical		0.005	0.025
*Pericopsis laxiflora* (Benth. ex Baker)Meeuwen	Leguminosae	Kodoléya	TOGO15411	Ro, Le, Ba, Fr	Powder	Topical, Oral			0.015
*Phyllanthus muellerianus* (Kuntze) Exell	Euphorbiaceae	Limbré Limbré	TOGO15412	Root, Leaves, Seed	Powder	Oral		0.005	0.010
*Piper guineense* Thonn.	Piperaceae	Djéyawa	TOGO15413	Seed	Powder, Decoction	Topical	0.010		0.015
*Prosopis africana* Taub.	Leguminosae	Kpalo	TOGO15414	Root, Bark, Leaves	Mashing	Oral		0.005	0.005
*Pseudocedrela kotschyi* Harms	Meliaceae	Btétéouré/ Hététéoudè	TOGO15415	Root, Bark, Leaves	Powder	Oral, Topical		0.015	0.005
*Pteleopsis suberosa* Engl. & Diels	Combretaceae	Sissinow/ Kizizina	TOGO15416	Root	Decoction	Oral, Topical	0.010		0.005
*Pterocarpus erinaceus* Poir*.*	Fabaceae	Tem/ Tem	TOGO15417	Leaves, Root	Maceration, Decoction, Infusion, powder	Oral		0.010	
*Rourea coccinea* (Schumach. & Thonn.) Hook.f.	Connaraceae	Tchamalido	TOGO15418	Root	Infusion, Dough	Oral, Topical			0.015
*Sarcocephalus latifolius (SM.)* E.A.Bruce	Rubiaceae	Kidjithilo/ Kakayo	TOGO15419	Root, Leaves, Bark, Whole plant, Fruit	Maceration, Infusion, Decoction, Powder	Oral, Topical	0.010	0.015	0.015
*Securidaca longepedunculata* Fresen.	Polygalaceae	Fozi/ Bnbna	TOGO15420	Root, Bark, Leaves, Whole plants	Dough, Decoction, Infusion	Oral, Topical	0.015	0.036	0.005
*Senna occidentalis (L.)* Lien	Caesalpiniaceae	Ktchintchin	TOGO15421	Leaves, Root	Powder	Topical			0.005
*Sida acuta* Burm.f.	Malvaceae	Nbazoudou/ Kpenzalo	TOGO15422	Leaves, Whole plant	Powder	Oral, Topical			0.010
*Syzygium aromaticum (L.)* Merr. & L.M.Perry	Myrtaceae	Kanafourou	TOGO15423	Root, Bark, Seed, Leaves	Decoction, Infusion	Oral	0.005		0.025
*Terminalia glaucescens* Planch. ex Benth.	Combretaceae	Souwo/ Simtéou	TOGO15424	Root, Leaves, Bark	Maceration	Topical			
*Trichilia emetica* Vahl	Meliaceae	Adjendjakpézou	TOGO15425	Root, Leaves, Bark	Infusion, Dough	Ocular, Oral, Topical		0.015	0.005
*Uvaria chamae* P.Beauv*.*	Annonaceae	Doumfodou	TOGO15426	Root	Maceration	Topical		0.005	0.005
*Xylopia aethiopica* A.Rich.*	Annonaceae	Soozi/ Koékrabi, Soossé	TOGO15427	Fruit	Decoction, Infusion, Powder	Oral, Topical	0.020	0.061	0.061

This table presents the used values (UV), administration route, formulation of drugs from each species of plants and the scientific, local name of each plant for the treatment of onchocerciasis, lymphatic filariasis, schistosomiasis, dermatitis and inflammation. * show the most cited plants by the THs for the treatment of helminthiasis and selected for in vitro screening

According to the UV score, *A. melegueta*, *X. aethiopica* and *K. senegalensis* were the most frequently used for the treatment of onchocerciasis, schistosomiasis and lymphatic filariasis*. A. melegueta* was the most cited by the THs for the treatment of onchocerciasis (UV = 0.036) while *X. aethiopica* was associated with the treatment of schistosomiasis (UV = 0.061) and lymphatic filariasis (UV = 0.061). Moreover*, A. melegueta*, *X. aethiopica* and *K. senegalensis* were also more used for the treatment of inflammation (UV = 0.066, UV = 0.056 and UV = 0.025 respectively, data not shown). The agreement among the THs for treating a given disease with plant materials was high with 0.57 for onchocerciasis, 0.66 for lymphatic filariasis and 0.61 for schistosomiasis.

**Fig. 3:**
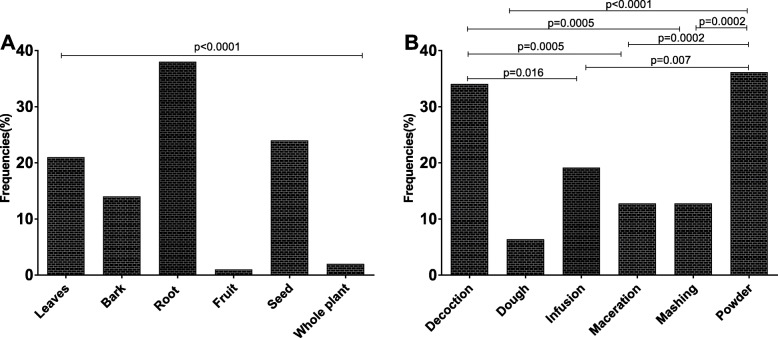
Preparation mode and plant’s organs used. Bars indicated the frequencies (%) of the mode of the preparation of the drug **a** and the parts of the plants used **b**. Chi-square was used to compare differences between groups and p value was less than 0.0001

Roots, seeds, leaves and barks were the main plant’s organs used by THs to prepare their medicinal recipes respectively, 35, 24, 21 and 14% (Fig. 3a). All the interviewed THs claimed to harvest plant materials during any season and at any time of the day.

For the formulation of medicinal recipes, powder (36.17%) and decoction (34.04%) were most cited (Fig. 3b). In addition, it was observed that most of the THs administrated the preparation by oral (51.06%) or topical (63.83%) routes.

### Anthelmintic effects

**Fig. 4:**
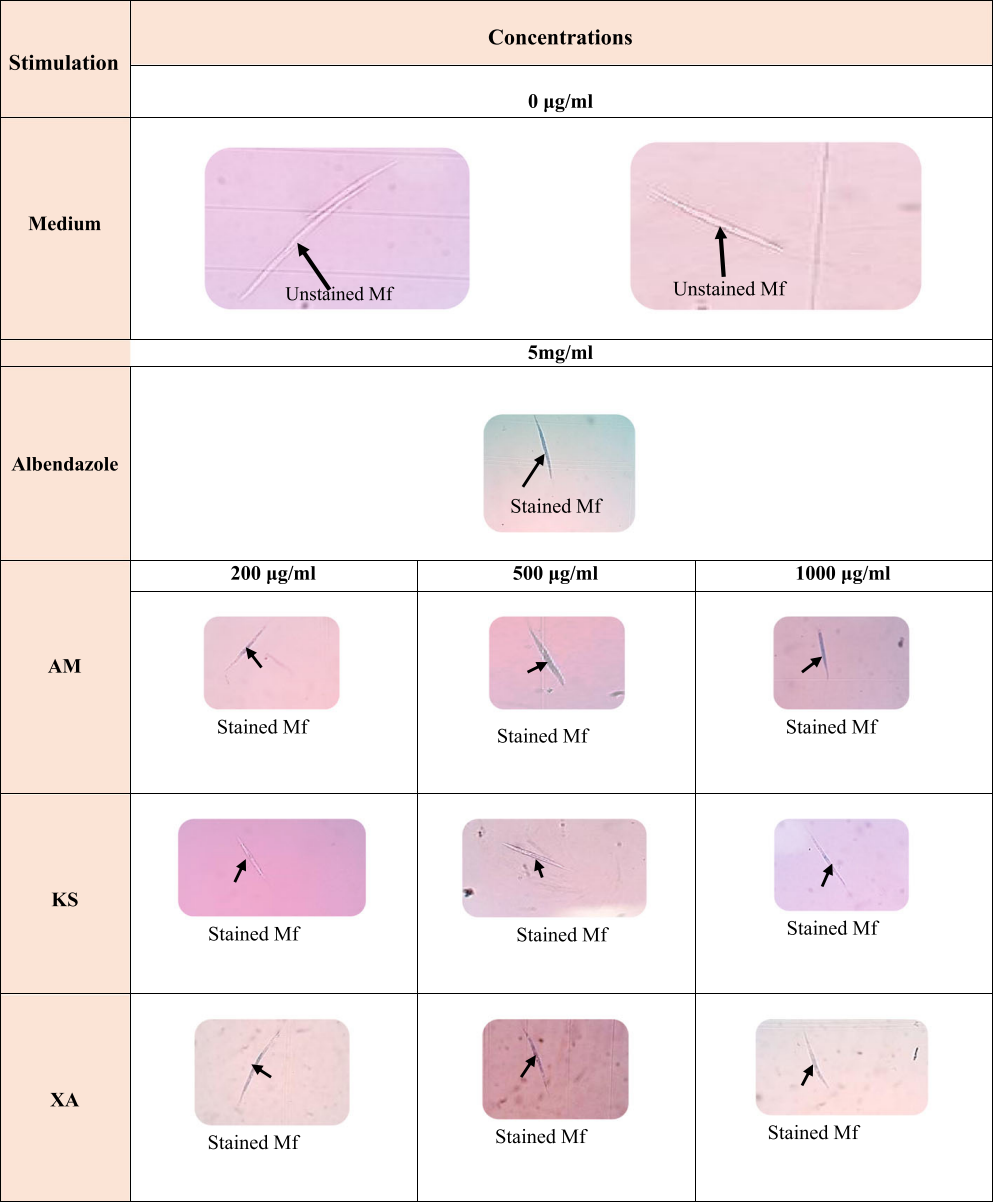
Anthelmintic effect of *Xylopia aetiopica* (XA), *Aframomum melegueta* (AM) and *Khaya senegalensis* (KS) on microfilariae (Mf) of *Litomosoides sigmodontis*. Mf (*n* = 75/well) of *Litomosoides sigmodontis* were cultured in absence of plant extracts (0 μg/ml, medium) or in presence of AM (green line), KS (blue line) and XA (red line) at different concentrations (200 μg/ml, 500 μg/ml and 1000 μg/ml). After 7 days culture, the viability of microfilariae were evaluated using typan blue. Graph shows the percentage of dead Mf for each concentration. The concentration that induced 50% of death was designed lethal dose 50 (LD50)

In the anthelmintic assay, the microfilariae from the control subculture, without drug were not stained, while microfilariae from Albendazole, *A. melegueta*, *K. senegalensis* and *X. aethiopica*, subculture were stained blue in the presence of trypan blue (Fig. 4). This indicated that *A. melegueta*, *K. senegalensis* and *X. aethiopica* had induced microfilariae death. The concentration that induced the death of 50% of microfilariae was 233 μg/mL for *X. aethiopica*, 265 μg/mL for *A. melegueta* and 550 μg/mL for *K. senegalensis* (Fig. 4).

### Cytotoxicity

**Fig. 5:**
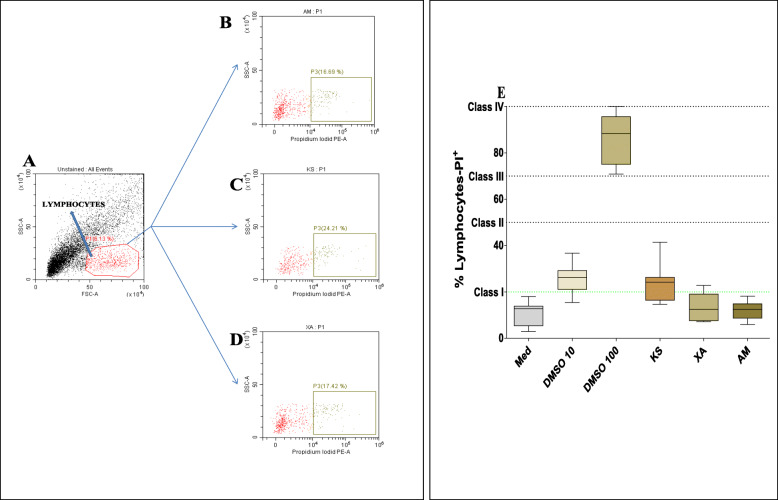
Cytotoxicity of the most frequently used plants by the THs for the treatment of helminthiasis: Human PBMCs (2 × 10^5^ cells/well) were left alone (Med) or stimulated with 200 μg/ml of AM, KS, XA, DMSO 100 and 10% for 24 h. Cells were stained with propidium iodide dye (PI) and acquired by flow cytometry. **a**: lymphocytes gate, **b**, **c**, and **d** are PI^+^ cells in presence of AM, KS and XA respectively. (E) Box whiskers (tukey) with outliers show the percentage of lymphocytes expressing PI (*n* = 13). *P* values were determined by Mann-Whitney U-test. The NF EN ISO 10993-5 standards classifications were indicated by class I (not cytotoxic), class II (moderate cytotoxicity), class III (Benign cytotoxicity) and class IV (severe cytotoxicity)

The cytotoxicity of the plants was evaluated by flow cytometry using PI staining after PBMCs were cultured with the plant extracts*.* According to the NF EN ISO 10993-5 standards classification, *A. melegueta* and *X. aethiopica* plant extracts were not cytotoxic. This was indicated by less than 20% of CD4^+^PI^+^cells. It was however observed that *K. senegalensis* induced moderate cell toxicity (24 ± 8% of CD4^+^PI^+^ cells) (Fig. 5).

## Discussion

This study aimed to document the use of plants in the treatment of helminthiasis, the survey revealed that male THs were more represented contrary to the reports of Gale et al. who found that women in Togo were more represented in complementary and alternative medicine [14]. The reason could be linked to cultural issues in the study region, since women retain a more traditional role as homeworkers. The findings however were in accordance with other study that showed the predominance of men among TH in *Tem* tribe [10]. The majority of THs were over 40 years old and had more than 10 years professional experience in traditional medicine. Most of them inherited their knowledge from their family confirming previous findings in this region [15]. To diagnose the disease, the THs rely on symptoms. Characteristic symptoms of lymphatic filariasis are hydrocele and lymphedema [16]. The diagnosis of urinary schistosomiasis should be especially suspected in cases of terminal hematuria and eosinophilia. It can be considered that the interviewed THs have a basic knowledge of the target diseases because they can recognize most of the specific symptoms [17].

Some plant families seem to stand out in any pharmacopoeia. A study on antimalarial plants in the Maritimes region of the same country revealed that out of 52 antimalarial plants species, Rubiaceae and Rutaceae were the most used to combat malaria [18]. In a study conducted in the central plate of Burkina Faso, the following families Caesalpiniaceae, Poaceae, Mimosaceae and Fabaceae have been ranked amongst the richest in species citations [19]. These medicinal plants were distributed among 28 families, the largest proportion belonging to the families Fabaceae and Anacardiaceae*.* Telefo et al. also identified 46 plant species belonging to 26 families, the largest number of species recorded in Asteraceae and Acanthaceae [20]. In this study, 41 species were recorded and the largest number of species belonged to Leguminosae and Annonacea. The preference for their use may be related to their accessibility, as they are common and grow more in this area*.* According to Heinrich et al., when the consensus factor of informants is high, it reflects a good knowledge of medicinal plants, a collective knowledge of their uses, but also an exchange of information between THs [21]. In the study the ICF was high meaning that there was a great agreement among the THs regarding the use of these plants for the treatment of helminthiasis. Many authors studied the anthelmintic properties of plants in Africa [22]. Several plants used in the treatment of helminthiasis were found active in in vitro screenings. For examples *Ceratonia siliqua* extract was shown to ameliorate *Schistosoma mansoni*-induced liver fibrosis, while *Verbascum sinaiticum* and *Commiphora swynnertonii* exerted trypanocidal activity [23, 24]. Anthelmintic effects of *A. melegueta* on *helminth parasites*, were observed by Akinsanya et al. [25]. The ethanolic extract of *A. melegueta* also known as “grain of paradise” has anti-inflammatory properties by inhibiting the activity of cyclooxygenase-2 (COX-2) enzyme [26]. *A. melegueta* seeds are also used in Africa to treat diarrhoea and gastroenteritis [27]. In southern Nigeria, Benin and Togo, it is employed in divine practices [28]. *Xylopia aethiopica* was mostly used for the treatment of filariasis and schistosomiasis [29]. The plant is also used commonly in Nigeria by traditional herbalists to treat gastrointestinal helminth parasites [30]. Ademola et al. suggested the use of *K. senegalensis* extract in anthelmintic therapy in veterinary practice [31]. Antitrypanosomal activity of *K. senegalensis* was also investigated by some authors [32]. In the present study, in vitro anthelmintic activities of the three plants was investigated using microfilariae of *Litomosoides sigmodontis* at different concentrations of the plant extracts. The mortality of Mf was dose-dependent and *X. aethiopica* had more effect on the microfilariae indicated by the lowest LD_50_. These data justify the use of the cited plants by the THs for the treatment of helminthiasis. Further studies on the effect of these plants on parasite paralysis and mobility should be carried on.

Fruit extracts are used to treat coughs, bronchitis, dysentery rheumatism and malaria [33–35]. The study showed that root, leaves and seed were the parts of the plants most cited by the THs for the treatment of helminthiasis. Previous studies have shown that leaves and roots were mostly used for the treatment of asthma [36]. A similar study showed that to treat liver disease, the most used parts of this plant were the leaves and roots, thus extracts of this plant can aid in a broad spectrum of symptoms [37].

The cytotoxicity of the three studied plants by the THs to treat helminthiasis was evaluated on human peripheral mononuclear blood cells (PBMCs) by flow cytometry. This method has the advantage to directly show the toxicity of the plant for human cells but an in vivo evaluation could indicate exactly which vital organs are damaged. *Afromomum melegueta* and *X. aethiopica* were not cytotoxic at 200 μg/mL but *K. senegalensis* revealed moderate toxicity with cell mortality above 20% at the same concentration. Many studies were performed on the cytotoxicity of the three extracts. Sahar et al*,* showed that *K. senegalensis* would be toxic to human liver, breast and colon cancer cells with IC_50_ of 61.1, 79.7, and 61 μg/mL respectively and sesquiterpens occurring in the plant would be responsible for its toxicity [38]. On the other hand, Idoh et al. revealed that *A. melegueta* has a hepatoprotective effect on rats [29]. Apart from its hepatoprotective property, *A. melegueta* would also have anti-apoptotic properties [39]. Similarly, volatile oil from *X. aethiopica* was found to be non-toxic to human epidermal cells line [40].

Many studies have demonstrated the anthelmintic effects of total phenolic and flavonoids compounds [41–44]. However, it would be difficult to say with accuracy, that these chemical groups are responsible for the activity observed in the present study. Thus, further bioguided fractionation of each plant should be undertaken to identify the active principles.

## Conclusion

This study demonstrated that THs had knowledge about the treatment of helminthiasis based on plants materials and highlighted the main plants used in the Central and Kara region of Togo. The anthelmintic and cytotoxicity effects of these commonly used plants were delineated. The benefits of listening to THs in such endemic areas is paramount to unveil potential new sources for fighting helminthiasis in general and moreover, contribute to the identification of new molecules for the treatment of symptoms and conditions arising from chronic helminth infections.

## Data Availability

The datasets during and/or analysed during the current study available from the corresponding author on reasonable request.

## Abbreviations

AM: *Aframomum melegueta*
CD4: Cluster of Differenciation Antigen 4
CERMETRA: Centre d’Etude et de Recherche en Médecine Traditionnelle Appliquée du Togo
CO_2_: Carbon Dioxide
COX-2: Cyclooxygenase-2
DALYs: Disability-Adjusted Life Years
DMSO: Dimethyl Sulfoxide
EN: European Norm
FCS: Fetal Calf Serum
IC50: The half maximal inhibitory concentration
ICF: Informant Consensus Factor
IPNI: International Plant Names Index
ISO: International Organization for Standardization
KS: *Khaya senegalensis*
LD50: Median Lethal Dose
LF: Lymphatic filariasis
Mf: Microfilariae
NF: Norme Française
NGO: Non-Governmental Organization
Nt: Number of species used in the treatment of a given disease
Nuc: Number of citations for the treatment of a given disease
PBMCs: Peripheral Blood Mononuclear Cells
PBS: Phosphate-buffered saline
PI: Propidium Iodide
RPMI: Roswell Park Memorial Institute medium
THs: Traditional Healers
UV: Used Value
XA: *Xylopia aethiopica*
CBRS: Comité de Bioéthique pour la Recherche en Santé
